# Use of a Deep Recurrent Neural Network to Reduce Wind Noise: Effects on Judged Speech Intelligibility and Sound Quality

**DOI:** 10.1177/2331216518770964

**Published:** 2018-04-30

**Authors:** Mahmoud Keshavarzi, Tobias Goehring, Justin Zakis, Richard E. Turner, Brian C. J. Moore

**Affiliations:** 1 Department of Psychology, University of Cambridge, Cambridge, UK; 2 MRC Cognition and Brain Sciences Unit, University of Cambridge, Cambridge, UK; 3 Blamey and Saunders Hearing Pty Ltd, East Melbourne, Victoria, Australia; 4 Department of Engineering, University of Cambridge, Cambridge, UK

**Keywords:** hearing aids, wind noise, speech intelligibility and sound quality, machine learning, neural networks

## Abstract

Despite great advances in hearing-aid technology, users still experience problems with noise in windy environments. The potential benefits of using a deep recurrent neural network (RNN) for reducing wind noise were assessed. The RNN was trained using recordings of the output of the two microphones of a behind-the-ear hearing aid in response to male and female speech at various azimuths in the presence of noise produced by wind from various azimuths with a velocity of 3 m/s, using the “clean” speech as a reference. A paired-comparison procedure was used to compare all possible combinations of three conditions for subjective intelligibility and for sound quality or comfort. The conditions were unprocessed noisy speech, noisy speech processed using the RNN, and noisy speech that was high-pass filtered (which also reduced wind noise). Eighteen native English-speaking participants were tested, nine with normal hearing and nine with mild-to-moderate hearing impairment. Frequency-dependent linear amplification was provided for the latter. Processing using the RNN was significantly preferred over no processing by both subject groups for both subjective intelligibility and sound quality, although the magnitude of the preferences was small. High-pass filtering (HPF) was not significantly preferred over no processing. Although RNN was significantly preferred over HPF only for sound quality for the hearing-impaired participants, for the results as a whole, there was a preference for RNN over HPF. Overall, the results suggest that reduction of wind noise using an RNN is possible and might have beneficial effects when used in hearing aids.

## Introduction

Airflow around a hearing aid can lead to turbulence at the microphone ports. The resulting random fluctuations in air pressure are often referred to as wind noise (Chung, Mongeau, & McKibben, 2009; Zakis, 2011). This noise can be disturbing in some conditions, such as when outdoors on a windy day, riding a bicycle, or running. Wind noise can have strong deleterious effects on the perception of speech and other sounds because its level can be as high as 85 dB SPL for a wind speed of 3 m/s and 100 dB SPL for a wind speed of 6 m/s (Zakis, 2011). Wind noise levels reach a maximum of about 110 to 115 dB SPL for a wind speed of 12 m/s, due to microphone saturation (Zakis, 2011). A survey by Kochkin (2010) indicated a 42% dissatisfaction rate among hearing aid users with the performance of their hearing aids in windy environments.

Wind noise varies with wind angle, microphone location, and across and within styles of hearing aids (Chung et al., 2009; Zakis, 2011). For moderate wind velocities, the energy in wind noise is mainly concentrated at frequencies below about 300 Hz, the spectrum level decreasing at a rate of about 26 dB/oct for frequencies above that (Korhonen, Kuk, Seper, Morkebjerg, & Roikjer, 2017; Raspet, Webster, & Dillion, 2006; Wutte, 1992). As the wind velocity increases, the spectrum of the wind noise spreads toward higher frequencies (Chung et al., 2009; Korhonen et al., 2017; Zakis, 2011). It remains one of the main challenges for hearing aid manufacturers to improve the perception of speech with hearing aids in windy environments.

Amelioration of the effects of wind noise in hearing aids is based on two approaches: acoustic design modifications and signal processing (Korhonen et al., 2017). The first approach involves mechanical changes to the design of hearing aids, such as placing the microphone in the small indentation between the crura and the antihelix to decrease turbulence, adding a cover on top of the microphone to diffuse the wind flow, and putting a piece of foam on top of the microphone to reduce the wind velocity (Kates, 2008).

The second approach uses digital signal processing techniques to determine whether wind noise is present and to reduce wind noise when it is detected. Detection of wind noise is usually based on the fact that it is largely uncorrelated at the two (or more) microphones of a hearing aid, despite the microphones being located close to one another (Kates, 2008), whereas input signals such as speech or music produce more highly correlated outputs from the microphones. Hence, wind noise can be detected as a decorrelation of the two microphone signals (Kates, 2008; Zakis & Tan, 2014). The reduction of wind noise is often based on a simple attenuation of the signal at low frequencies. For hearing aids with only one microphone, signal-processing approaches include dictionary-based sparse coding (Schmidt, Larsen, & Hsiao, 2007) and noise reduction with adaptive post filtering (Nemer & Leblanc, 2009).

In the past few years, Machine Learning (ML) has been widely used to achieve substantial improvements in computational tasks in the visual, auditory, speech, and language domains. A major advance came from the use of artificial neural networks with more than two layers (Hinton, Osindero, & Teh, 2006). These can generally be categorized into three main architectures: feed-forward deep neural networks (DNN), recurrent neural networks (RNN), and convolutional neural networks (CNN). In comparison to simple DNNs, CNNs have been used as a more powerful class of model for recognizing visual content in tasks like image recognition, segmentation, detection, and retrieval (Karpathy et al., 2014). However, RNNs have achieved superior results for the detection and recognition of temporal patterns in speech, language, and time series data by making use of the interdependence of data samples across time (Graves, Mohamed, & Hinton, 2013; Lipton, Berkowitz, & Elkan, 2015). One of the most successful RNN architectures is the long short-term memory (LSTM) model proposed by Hochreiter and Schmidhuber (1997). It was designed to model the long-range dependencies of temporal sequences in a more accurate way than with conventional RNNs (Sak, Senior, & Beaufays, 2014).

Several ML-based approaches using DNNs to segregate speech from masking noise have been shown to improve speech perception in noise for normal-hearing and hearing-impaired listeners and users of cochlear implants (Chen, Wang, Yoho, Wang, & Healy, 2016; Goehring et al., 2017; Healy, Yoho, Chen, Wang, & Wang, 2015; Healy, Yoho, Wang, & Wang, 2013; Monaghan et al., 2017; Tchorz & Kollmeier, 2003). Recently, the use of RNNs for segregation of speech from noise had led to improvements in estimation accuracy and generalization performance compared with DNN-based methods (Chen & Wang, 2017; Huang, Kim, Hasegawa-Johnson, & Smaragdis, 2015; Kolbæk, Yu, Tan, & Jensen, 2017; Weninger et al., 2015). ML-based approaches have been successfully applied to acoustic conditions with nonstationary noise maskers at low signal-to-noise ratios, and thus promise to be good candidates for the reduction of wind noise, although to our knowledge, they have not yet been evaluated with this application in mind. So far, RNN-based methods have been shown to improve performance over DNN-based methods when using computational measures of quality and intelligibility, but the benefits for speech perception in noise by human listeners remain unclear.

This article reports a study of wind noise reduction using an ML algorithm based on a deep (multilayer) LSTM model. For brevity, this is referred to hereafter simply as the RNN. The RNN was first trained to predict the ideal ratio mask (IRM, described in detail later), using recordings of speech corrupted by wind noise. The clean (noise-free) speech was used to estimate the target IRM. Then, the trained model was used to process the noisy speech, so as to attenuate time-frequency segments with low speech-to-noise ratio (SNR) while retaining segments with high SNR. The effects of the algorithm on judged speech intelligibility and sound quality were assessed for speech signals affected by wind noise, recorded using the microphones of behind-the-ear (BTE) hearing aids. Simple high-pass filtering was used for a comparison condition.

## Method

### Ethical Approval

The study was approved by the Psychology Research Ethics Committee of the University of Cambridge and was conducted in accordance with the Declaration of Helsinki.

### Participants

Eighteen native English-speaking participants took part in the experiment. Audiometric thresholds were measured for audiometric frequencies from 0.25 to 8 kHz, using a Grason-Stadler GSI-61 audiometer. Nine of the participants had normal hearing (5 female, average age 31 years), with audiometric thresholds lower than 20 dB HL for all measured frequencies, and nine had hearing loss. Only the better-hearing ear of each participant was tested (based on the average threshold across 0.5 to 4 kHz). The sex, age, and audiometric thresholds for the test ear of the hearing-impaired participants are shown in Table 1. Seven of the hearing-impaired participants were users of hearing aids. The experiment lasted about 2 hours for each participant, and participants were paid for taking part as well as receiving reimbursement for travel expenses.

**Table 1.** Age, Sex, and Audiometric Thresholds (in dB HL) of the Test Ears of the Hearing-Impaired Participants.

	Age (years)	0.125	Frequency, kHz							
Sex			0.5	1	2	3	4	6	8
Male	73	10	20	25	40	50	55	50	70
Male	61	15	20	30	35	55	50	40	25
Female	46	5	20	30	45	45	50	30	30
Male	71	15	20	20	10	30	45	50	60
Female	45	5	10	10	25	40	30	25	10
Male	62	15	30	20	40	55	50	55	55
Male	77	10	5	5	25	45	70	65	65
Female	66	15	20	15	30	35	40	40	55
Female	53	5	10	5	30	25	45	40	25

### Recording Procedure

**Figure 1.**
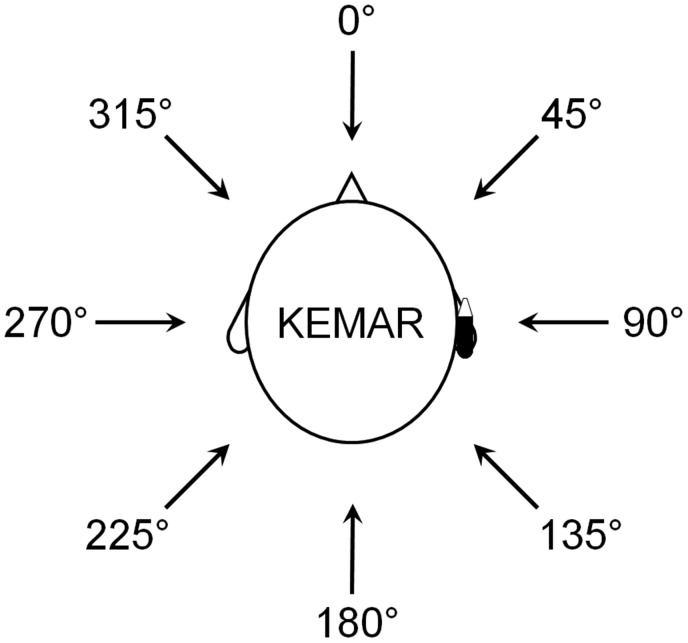
Diagram of KEMAR dummy head with a hearing aid on the right ear and different azimuth angles used for presenting wind and speech signals.

The wind and speech azimuths were defined based on the KEMAR dummy head (Burkhard & Sachs, 1975) as shown in Figure 1; 0° was in front of the head, 90° was to the right side of the head on the side where the hearing aid was placed, 270° was to the left side of the head, and 180° was at the back of the head. The wind noise was produced by turbulence around the microphone ports of the hearing aid, using a low-noise wind source, as described by Zakis (2011). Adobe Audition 1.5 computer software (Adobe, San Jose, CA) was used to collect 32-bit recordings at a sampling rate of 44.1 kHz for both front and rear microphones of the BTE hearing aid and saved as stereo WAV files. Recordings were 20-seconds long, the first 10 seconds consisting of wind noise plus simultaneously presented male speech (continuous reading of a story, taken from track 3 of CD 2 described by Keidser et al., 2002) and the second 10 seconds consisting of wind noise plus simultaneously presented female speech (CUNY sentence lists). The wind speed was 0 (producing no wind noise), 3, 6, and 12 m/s. However, the speech-to-wind-noise ratio was very low for the two highest wind speeds, so the present work is based on recordings obtained using the 0 - and 3 -m/s wind speeds. The wind noise for the 3 -m/s wind speed was clearly audible and intrusive. Its overall level was typically about 80 dB SPL but was as high as 85 dB SPL for some azimuths. Wind azimuths ranged from 0 to 315° in 45° steps. The speech was presented at 70 dB SPL from a loudspeaker that was 1 m from the KEMAR and at an azimuth that was 45° clockwise from that of the wind. The hearing aid shells were “BTE1” (very large, with earhook) and “BTE2” (medium size, with earhook), as described by Zakis (2011).

Initially, the recorded signals were down-sampled to 22.05 kHz. The two microphones output signals were considered as:

(1) $$x_{1}(t)=s(t)+v_{1}(t)$$

(2) $$x_{2}(t)=s(t-{\text{t}}_{\text{d}})+v_{2}(t)$$

where *s* is the clean speech, *x_i_* and *v_i_* (*i* = 1, 2) are the noise-corrupted speech and the wind noise for the *i*th microphone, respectively, and t_d_ is the time delay between the microphones.

### ML Algorithm

**Figure 2.**
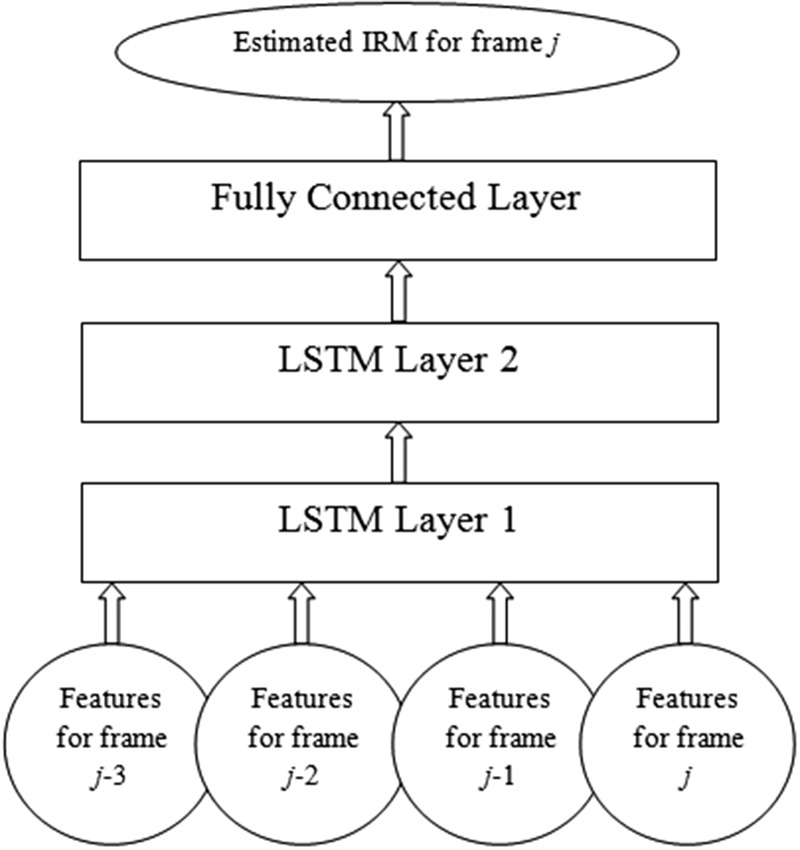
Overview of the LSTM network used in this study.

As described in the Introduction section, the LSTM model variant of the RNN proposed by Hochreiter and Schmidhuber (1997) was used in the present work. The LSTM model maps sequential input vectors to sequential vectors of outputs using iterative equations (Graves et al., 2013). In this study, an RNN was constructed that consisted of an input layer, two LSTM layers with 96 units followed by a fully connected layer with 64 units, and an output layer (as shown in Figure 2). The number of units in the input and output layers of the RNN was defined by the input feature and output mask dimensions, respectively. The RNN processed a four-time-step input and each step corresponded to features extracted from each single frame of speech; Steps 1, 2, 3, and 4 corresponded to successive frames *j*-3, *j*-2, *j*-1, and *j*, respectively. The RNN took acoustic features as its inputs and predicted the IRM (Delfarah & Wang, 2017; Healy, Delfarah, Vasko, Carter, & Wang, 2017), based on the ideal Wiener filter in the time-frequency (T-F) domain. The IRM for the *j*th frame and *m*th channel was defined as follows (Delfarah & Wang, 2017):

(3) $$\mathrm{IRM}(j,m)=\sqrt{\frac{S_{i}^{2}(j,m)}{S_{i}^{2}(j,m){+V}_{i}^{2}(j,m)}}$$

where index *i* refers to the microphone number. *S_i_*(*j, m*) and *V_i_*(*j, m*) represent the magnitudes of s*_i_*(*t*) and v*_i_*(*t*) in the *m*th channel of frame *j*, respectively. Note that the clean speech recordings were used to obtain s*_i_*(*t*). The microphone signals were segmented into frames with a duration of 5 ms (110 samples) and an overlap of 50% (55 samples) between successive frames. We used a 32-channel gammatone filter bank (Patterson, Allerhand, & Giguère, 1995) with channels equally spaced on the ERB_N_-number scale (Glasberg & Moore, 1990) and with center frequencies ranging from 50 to 11025 Hz to calculate the IRM in the time-frequency domain. Thus, the RNN output dimension was 64 (32 channels × 2 microphones).

Two groups of features were extracted from each of the gammatone-filtered outputs of the two microphone signals and used for training the network: gammatone features and correlation features. The gammatone features were the overall energy in each time-frequency unit. They provided perceptually relevant information about the short-term spectrum of the stimuli and changes in short-term spectrum over time. The gammatone features were normalized to have zero mean and unit variance. The correlation features were designed to exploit the fact that wind noise is largely uncorrelated at the outputs of the two microphones in a hearing aid, while the target speech is more highly correlated. The correlation features were calculated by applying the normalized cross-correlation operation to the gammatone filterbank outputs for the two microphone signals. The normalized cross-correlation for correlation lag *l* is given by:

(4) $$C(l,f)=\frac{r_{g_{1f}g_{2f}}(l,f)}{\sqrt{r_{g_{1f}g_{1f}}(0)r_{g_{2f}g_{2f}}(0)}};f=1,\ldots ,32$$

(5) $$r_{g_{1f}g_{2f}}(l,f)=\sum_{k=-N+1}^{N}g_{1f}(k-l)g_{2f}(k);f=1,\ldots ,32$$

(6) $$\begin{array}{c} r_{g_{\mathrm{if}}g_{\mathrm{if}}}(l,f)=\sum_{k=-N+1}^{N}g_{\mathrm{if}}(k-l)g_{\mathrm{if}}(k);\\ f=1,\ldots ,32,i=1,2 \end{array}$$

where *g*_1_*_f_* and *g*_2_*_f_* are samples obtained from filtering each frame of speech using the *f*th gammatone filter for microphones 1 and 2, respectively, *r_g_*_1_*_fg_*_2_*_f_* is the cross-correlation between *g*_1_*_f_* and *g*_2_*_f_*, *r_gifgif_* is the autocorrelation of *g_if_*, *i* is the microphone number, *l* is the correlation lag, and *N* is the number of samples in each frame. In this study, the correlation features were calculated with the lag set to 0, since the time delay between the two microphones was very small. There were 32 correlation features per frame. Accordingly, the total number of features for each time step of the RNN was 96, consisting of 32 gammatone features for each microphone and 32 correlation features.

To ensure that the RNN was tested only using previously unseen data, the data for the test conditions were excluded from the training data. This is called the “holdout cross-validation method.” The test conditions were chosen so that the speech came from the front or the side, as is usually the case in everyday life. The azimuths used for testing were as follows: wind at 0° with speech at 45° (0°, 45°), wind at 45°with speech at 90° (45°, 90°), and wind at 315° with speech at 360° (315°, 360°); note that in the last condition, the speech came from in front, as 360° is equivalent to 0°. In all cases, the test data were recordings from BTE1. The total potential set of training data consisted of 320 seconds of male and female speech corrupted by 3 m/s wind noise, as received by the two BTE microphones for all eight pairs of wind and speech azimuths from (0°, 45°) to (315°, 360°) and for both BTE1 and BTE2 (16 20-second sets of two microphone recordings). The training data actually used were based on 13 of the 16 sets (excluding recordings from BTE1 for the three pairs of test azimuths), giving 260 seconds of training data.

The freely available ML frameworks TFlearn and Tensorflow, written in the Python programming language, were used to construct, train, and test the RNN (Abadi et al., 2016; Tang, 2016). We used “RMSprop” (Riedmiller & Braun, 1993), an improved version of the resilient backpropagation algorithm for batch training, as the optimizer function in the training algorithm. The learning rate was initialized to 0.001 and decreased by a factor of 0.999 in each epoch (an epoch is a training run based on using all of the training data once). The batch size was 100 and the training algorithm was run for 100 epochs. The mean square error was utilized as the loss function for the optimizer. The accuracy of the RNN was assessed using a measure similar to the detectability index *d*′ (Green & Swets, 1974); see the Results section and Table 2 for details. The accuracy did not improve systematically after about 90 epochs, so training was deemed to be complete after 100 epochs. Once the RNN had been trained, the estimate of the IRM for each frame was used to process the speech signal corrupted by wind noise for that frame in the T-F domain so as to obtain an approximation to the clean speech signal. The processed frames were combined with the noisy phase information and the overlap-add operation was used to reconstruct the acoustic output signals. The noise-reduction processing with the ML algorithm was applied separately to the signals from each microphone, and then the two processed signals were summed to obtain a single output.

**Table 2.** Hit and False Alarm Rates (%) and *d*′ Values for the Estimated IRM.

Wind/speech angle	Hit rate (%)	False alarm rate (%)	*d*′
0°, 45°	78.9	1.9	2.87
45°, 90°	91.9	3.3	3.23
315°, 360°	80.6	2.0	2.91

### High-Pass Filtering Comparison Condition

Since wind noise is dominated by low-frequency components for moderate wind speeds, one way to reduce wind noise is to attenuate the low-frequency components of the microphone outputs. Indeed, this resembles the method of wind-noise reduction that is used in many hearing aids. In this study, we used a time-invariant high-pass filter as a simple simulation of this type of signal processing. For the wind speed used in our study, 3 m/s, the spectrum of the wind noise fell mainly below 500 Hz (Zakis, 2011). Hence, a steep finite impulse response high-pass filter with 513 taps and a cutoff frequency of 500 Hz was applied to the sum of the microphone signals. The filter was designed and implemented using the fir2 function in MATLAB and its relative response was 0 dB above 500 Hz and reached the −50-dB point at 410 Hz. This filter was steeper than the filters that are typically used in hearing aids, so it would have reduced the low-frequency noise more effectively than would be the case for most hearing aids. The high-pass filtering effectively removed frequency components in the speech below 500 Hz, which provide about 15% of the information in normal conversation speech (American National Standards Institute, 1997), but these components would, in any case, have been largely masked by the wind noise.

### Test Signals and Conditions

The participants were seated in a soundproof room and wore Sennheiser HD580 headphones connected to the sound card of a computer (with 24 bit resolution and a sampling rate of 22050 Hz). The root-mean-square input level (before frequency-dependent amplification for the hearing-impaired participants) of the speech (excluding the wind noise) was 60 dB SPL. Three conditions were used: noisy speech with no processing (NS), speech processed using the RNN, and high-pass filtered speech (HPF). The stimuli for the hearing-impaired participants were processed using linear frequency-dependent amplification according to the “Cambridge formula” (Moore & Glasberg, 1998) to ensure that the speech was audible over a wide frequency range. This was done using a 513-tap finite impulse response filter implemented using the fir2 function in MATLAB.

### Procedure

The three conditions were compared in terms of subjective intelligibility and sound quality, using a paired-comparison procedure. There were three types of paired comparisons: NS versus RNN, RNN versus HPF, and NS versus HPF. The procedure was the same as described by Moore and Sek (2013). The two sounds to be compared were presented in succession with a 200-ms silent interval between them. The possible orders were used equally often and the order was randomized across trials.

The main experiment consisted of two parts. In the first part, participants were asked to indicate their preference in terms of subjective intelligibility. For this part, the instructions to the participant, which appeared on the computer screen, were as follows: “On each trial you will hear the same sentence twice in succession. Please decide whether the first or second sentence is more intelligible and by how much, by using the mouse to position the slider on the screen.”

In the second part, participants indicated their preferences in terms of subjective sound quality and comfort. The instructions for this part were as follows: “On each trial you will hear the same sentence twice in succession. Please decide whether the first or second sentence is more comfortable and by how much, by using the mouse to position the slider on the screen.”

On each trial, each pair of sounds was presented only once. Participants responded using a mouse to select the position of a slider on the screen along a continuum labeled “1 much better,” “1 moderately better,” “1 slightly better,” “equal,” “2 slightly better,” “2 moderately better,” and “2 much better.” Choices were not restricted to the labeled points; any point along the slider could be chosen. Within a given block of trials, each of the three pairs of conditions was presented twice in both orders for each of six sentences (three for the female talker and three for the male talker), so there were 72 trials in a block. There were six blocks per participant (three blocks for paired comparisons related to subjective intelligibility and three blocks for paired comparisons related to sound quality).

Preference scores for each participant and each pair of conditions were computed in the following way. The extreme positions of the slider were arbitrarily assigned values of −3 and + 3. Regardless of the order of presentation in a given trial (Condition X first or Condition Y first), if X was preferred, the slider position was coded as a negative number and if Y was preferred, the slider position was coded as a positive number. For example, if the order on a given trial was Y first and X second, and the participant set the slider position midway between “2 slightly better” and “2 moderately better,” the score for that trial was assigned a value of –1.5. The overall score for a given comparison and a given azimuth was obtained by averaging the scores for the two orders for that comparison and azimuth for each participant. Scores were then averaged across participants, but separately for the normal-hearing and hearing-impaired participants. Therefore, preference scores were constrained to fall in the range −3 to + 3.

## Results

### Simulation Results for RNN Processing

To evaluate the accuracy of the RNN in estimating the IRM from the noisy speech signal, the estimated SNR for each time-frequency segment of the estimated IRM was compared with that for the IRM estimated using the clean speech signal. A classification threshold of 0 dB SNR was used to convert the IRM estimated by the RNN into a binary mask for the calculation of hit and false alarm rates (Goehring et al., 2017; Kim, Lu, Hu, & Loizou, 2009). The hit rate was defined as the percentage of correctly classified speech-dominated T-F units in the estimated IRM and the false alarm rate was defined as the percentage of noise-dominated T-F units incorrectly classified as speech-dominated T-F units in the estimated IRM. The detectability index *d*′ (Green & Swets, 1974) was calculated from the hit and false-alarm rates (see Table 2). The *d*′ values were high (close to 3) for all three test directions used, indicating high classification accuracy.

### Preferences Scores for Intelligibility

To assess whether the preference scores were significantly different from zero (indicating a significant preference for one processing method relative to another), the scores for each participant were first averaged across azimuths. This was considered to be reasonable since the spectrum and level of the wind noise differed for each azimuth. Then, Wilcoxon nonparametric tests were used to assess whether the mean of the nine resulting scores for each pair of conditions was significantly different from zero (using two-tailed tests). The *W* statistic was used, since the number of scores was small.

**Figure 3.**
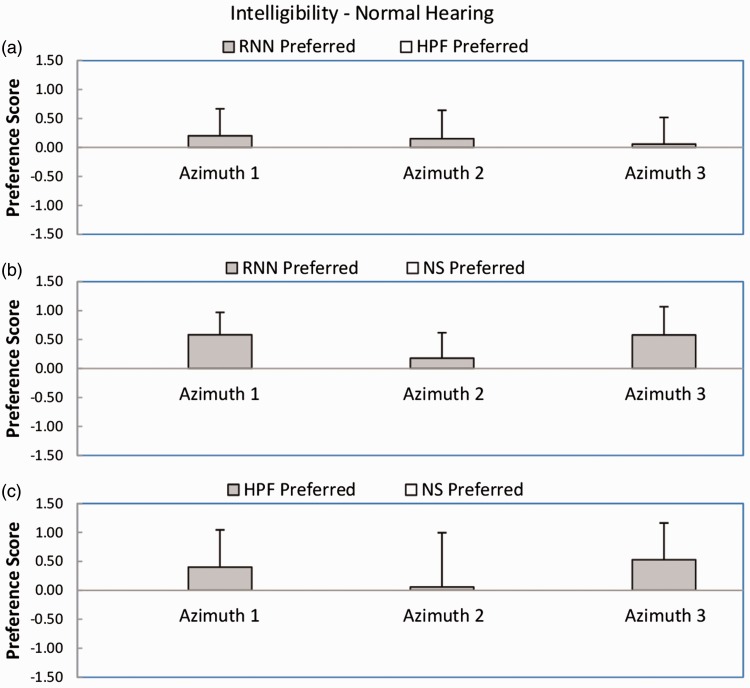
Preference scores for intelligibility for each azimuth and each pairwise comparison for the normal-hearing impaired participants. Each panel shows results for a different comparison, as indicated in the key: RNN = recurrent neural network; HPF = high-pass filtering; NS = noisy speech with no processing. Azimuths (wind, speech) 1–3 were: (0°, 45°), (45°, 90°), and (315°, 360°), respectively. Error bars show ± 1 standard deviation across participants.

Figure 3 shows mean preference scores and standard deviations for judged intelligibility for the normal-hearing participants for each azimuth and each pairwise comparison. For the comparison RNN versus HPF (Panel a), the mean preference scores were very slightly positive for all azimuths, favoring the RNN. However, the outcome of the Wilcoxon test was not significant (*W* = 15). For the comparison RNN versus NS (Panel b), the mean preference scores were all positive, favoring the RNN. The outcome of the Wilcoxon test was significant (*W* = 2, *p* < .05). For the comparison HPF versus NS (Panel c), the mean preference scores were positive for two azimuths and close to zero for the other. The outcome of the Wilcoxon test was not significant (*W* = 12). In summary, RNN was significantly preferred over NS, but HPF was not significantly preferred over NS. There was no significant difference between preferences for RNN and HPF.

**Figure 4.**
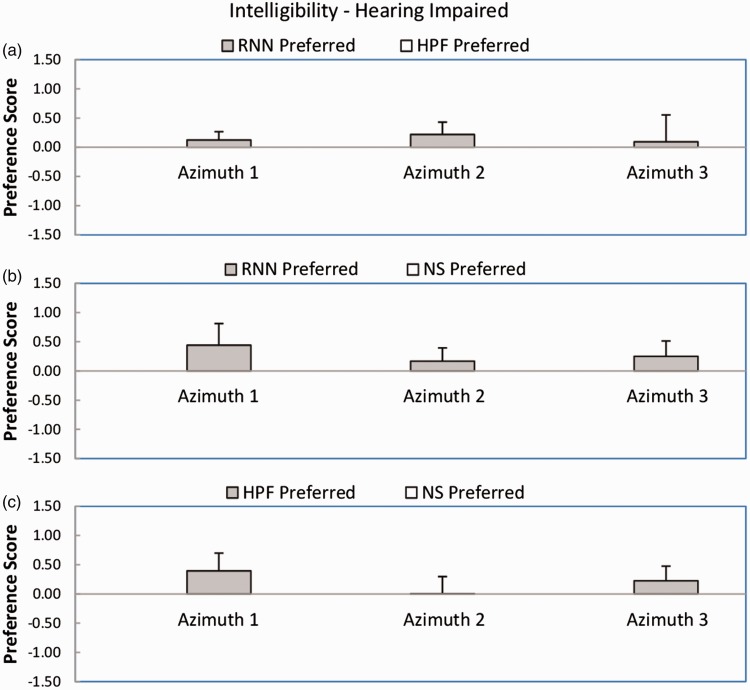
As Figure 3, but for the hearing-impaired participants.

Figure 4 shows mean preference scores and standard deviations for intelligibility for the hearing-impaired participants. The pattern of the results was broadly similar to that for the normal-hearing participants. For the comparison RNN versus HPF (Panel a), the mean preference scores were positive for all azimuths, favoring the RNN, but the outcome of the Wilcoxon test was not significant (*W* = 6). For the comparison RNN versus NS (Panel b), the mean preference scores were all positive, favoring the RNN. The outcome of the Wilcoxon test was significant (*W* = 0, *p* < .05). For the comparison HPF versus NS (Panel c), two out of three preference scores were positive, favoring the HPF, but the score for azimuth 2 was very close to zero. The outcome of the Wilcoxon test was not significant (*W* = 6). In summary, RNN was significantly preferred over NS, but HPF was not significantly preferred over NS. There was no significant difference between preferences for RNN and HPF.

### Preferences Scores for Quality

**Figure 5.**
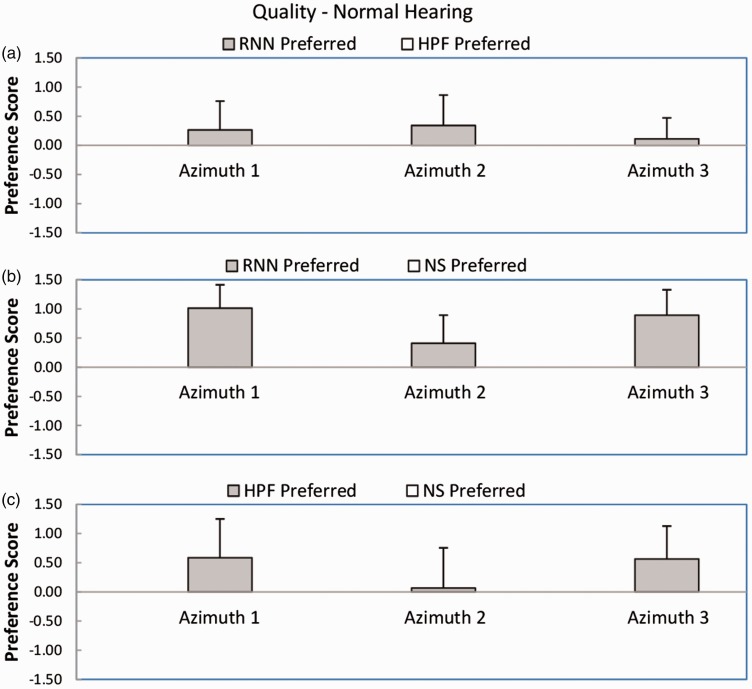
As Figure 3, but for sound quality or comfort.

Figure 5 shows mean preference scores and standard deviations for sound quality or comfort for the normal-hearing participants. For the comparison RNN versus HPF (Panel a), the mean preference scores were positive for all azimuths, favoring the RNN. However, the outcome of the Wilcoxon test was not significant (*W* = 11). For the comparison RNN versus NS (Panel b), the mean preference scores were all positive, favoring the RNN. The outcome of the Wilcoxon test was significant (*W* = 0, *p* < .05). For the comparison HPF versus NS (Panel c), two out of three preference scores were positive, favoring the HPF, but the score for azimuth 2 was very close to zero. The outcome of the Wilcoxon test was not significant (*W* = 8). In summary, RNN was preferred over NS, but HPF was not significantly preferred over NS. There was no significant difference between preferences for RNN and HPF.

**Figure 6.**
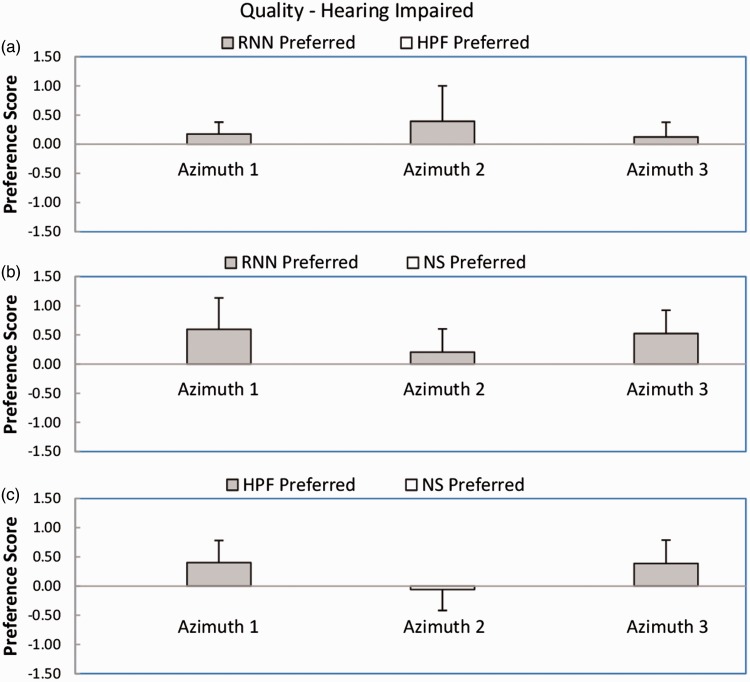
As Figure 5, but for the hearing-impaired participants.

Figure 6 shows mean preference scores and standard deviations for sound quality or comfort for the hearing-impaired participants. For the comparison RNN versus HPF (Panel a), the mean preference scores were positive for all azimuths, favoring the RNN. The outcome of the Wilcoxon test was significant (*W* = 3, *p* < .05). For the comparison RNN versus NS (Panel b), the mean preference scores were all positive, favoring the RNN. The outcome of the Wilcoxon test was significant (*W* = 0, *p* < .05). For the comparison HPF versus NS (Panel c), two out of three preference scores were positive, favoring the HPF, but the score for azimuth 2 was very slightly negative. The outcome of the Wilcoxon test was not significant (*W* = 6). In summary, RNN was preferred over NS and HPF, but HPF was not significantly preferred over NS.

## Discussion

The results showed small but significant preferences for RNN over NS for both subjective intelligibility and sound quality and for both groups. HPF was not significantly preferred over NS. Although there was only one significance difference between preferences for RNN and HPF in the individual Wilcoxon tests, the mean preference judgments for RNN versus HPF were positive for all azimuths for both groups for both subjective intelligibility and quality (12 cases), which is significant based on a binomial test (*p* = .0002). Thus, for the results as a whole, there was a preference for RNN over HPF.

The preferences were generally small. This probably reflects the fact that both signal processing methods reflect a trade-off between different factors. The RNN processing resulted in a reduction of wind noise, but at the expense of some audible artifacts; participants reported hearing some “gurgling” effects and they also reported that the noise was almost inaudible during brief pauses in the speech, while the noise was more audible when speech was present, so the noise appeared to fluctuate markedly. This probably happened partly because the RNN algorithm could accurately determine the SNR in T-F regions with very low SNR (i.e., T-F regions where the speech was essentially absent), but was less accurate when the SNR was intermediate. Also some of the artifacts probably arose from the use of the original noisy phase in reconstructing the signal, which would make the speech sound noisy even if the SNR were estimated perfectly. Differences in preference across participants may reflect differences in the way that they weight the benefits of RNN noise reduction against the deleterious effects of the artifacts. The artifacts could potentially be reduced by setting a limit to the attenuation applied by the RNN or by limiting the speed of the gain changes, as is done in the noise-reduction systems of some commercial hearing aids (Launer, Zakis, & Moore, 2016). This requires further research.

The HPF processing did not result in the type of artifacts that occurred for the RNN, but it had the effect of removing the lower frequency components in the speech, making the speech sound “thin” or lacking in bass. Moore and Tan (2003) reported that the “naturalness” of speech was markedly reduced by high-pass filtering at 313 Hz, and the high-pass cutoff frequency of 500 Hz used here would have had an even greater effect. Hence, the beneficial effects of noise reduction produced by the HPF were probably partly offset by the deleterious effects of removal of the low-frequency components of the speech. The less extreme forms of HPF that are used in commercial hearing aids could make speech sound less thin but at the cost of smaller beneficial effects.

It should be noted that the RNN used here operated “automatically.” Once trained, it was applied without any further adjustment of parameters. In principle, the RNN could be trained using recordings of wind noise obtained with a greater variety of wind speeds and azimuths and also with more different talkers than the two used here. Once trained, the RNN should automatically adjust its processing to deal with the changes in level and spectral content produced by differences in wind speed and azimuth, for example, produced by head movements. Furthermore, it should be possible to train the RNN so that it automatically does nothing when no wind noise is present. However, further work is needed to evaluate how well RNNs can be trained to generalize their performance to different wind speeds and talkers, including talkers not used for training.

While the high-pass filtering used here was found to be marginally effective, the HPF cutoff frequency was chosen to be appropriate for the wind speed of 3 m/s actually used in the experiment. To apply HPF processing in a real hearing aid, it would be necessary to have an additional algorithm for detecting the frequencies at which wind noise was present and a method of selecting the appropriate high-pass cutoff frequency.

The RNN processing was based on a relatively short frame duration of 5 ms, with a frame overlap of 50%. Ignoring limitations in signal-processing speed, the inherent delay produced by the RNN noise reduction would be about 7.5 ms, which is within the range that is acceptable for hearing aids (Stone & Moore, 1999, 2005). If the RNN processing were implemented in a hearing aid, it could be performed in parallel with the other processing performed in the hearing aid, and could even make use of the frequency analysis that is typically performed in hearing aids for other purposes, such as dynamic range compression, noise reduction, and directional processing (Launer et al., 2016). Hence, the RNN processing would not necessarily increase the time delay produced by the hearing aid.

Overall, the results suggest that processing using an RNN can be effective in reducing the effects of wind noise, and this has potential applications in hearing aids. Further research is needed to assess: (a) whether the benefits of RNN processing still occur when greater ranges of wind speeds and more talkers are used; (b) if better subjective intelligibility and quality are obtained when the amount and speed of wind noise reduction are limited to reduce artifacts; (c) if RNN processing is still beneficial when the RNN complexity is reduced to allow for the limited computational power of hearing aids.

## Summary and Conclusions

Two methods for reducing wind noise were evaluated. One method was a simple simulation of the processing that is often used in hearing aids and was based on HPF. The other method was based on the training of a RNN and the use of the trained network to reduce wind noise. The RNN was trained using recordings of the output of the two microphones of a BTE hearing aid in response to male and female speech at various azimuths in the presence of noise produced by wind from various azimuths with a velocity of 3 m/s, using the clean speech as a reference. A control condition using noisy unprocessed speech was also used (NS). A paired-comparison procedure was used to compare all possible combinations of the three conditions for subjective intelligibility and for sound quality or comfort. Eighteen native English-speaking participants were tested, nine with normal hearing and nine with mild-to-moderate hearing impairment. Frequency-dependent linear amplification was provided for the latter. Processing using the RNN was significantly preferred over no processing by both subject groups for both subjective intelligibility and sound quality, although the magnitude of the preferences was small. High-pass filtering was not significantly preferred over no processing. Although RNN was significantly preferred over HPF only for sound quality for the hearing-impaired participants, for the results as a whole, there was a preference for RNN over HPF. Overall, the results suggest that reduction of wind noise using an RNN is possible and might have beneficial effects when used in hearing aids.
